# Partial Epididymal Obstruction as a Cause of Idiopathic Oligozoospermia: A Reproductive Urologist’s Perspective Following 35 Years of Surgical and Clinical Experience

**DOI:** 10.3390/jcm13020382

**Published:** 2024-01-10

**Authors:** Giovanni M. Colpi, Ettore Caroppo

**Affiliations:** 1 Next Fertility Procrea, Andrology and IVF Center Unit, 6900 Lugano, Switzerland; 2 Asl Bari, Andrology Outpatients Clinic, 70014 Conversano (Ba), Italy; ecaroppo@teseo.it

**Keywords:** oligozoospermia, male infertility, epididymis, scrotal exploration, scrotal ultrasound, partial epididymal obstruction

## Abstract

The role of partial epididymal obstruction as contributing to the development of oligozoospermia has been neglected for decades. In the early 1970s, however, Robert Schoysman, a gynecological surgeon devoted to the surgical and medical management of male factor infertility, dedicated many efforts to study such a pathology and its possible effects on male fertility. Following the studies of this pioneer in the field, we concentrated our attention to the patterns of partial and complete epididymal obstruction during surgical scrotal exploration, once made possible even in oligozoospermic men by diagnostic and therapeutic interventions, such as vasovesciculography or seminal tract washout test, at present considered obsolete and no longer feasible in light of the current guidelines. Interestingly, we found signs of partial epididymal obstruction in about 30% of oligozoospermic men with normal testicular volume and serum FSH level as well as normal spermatogenesis at testis biopsy. We, then, compared the findings of scrotal ultrasound with those of scrotal exploration and found that the ultrasound abnormalities of the epididymis were highly predictive of anatomic alteration of the gland. In the present study, we report our experience, together with a historical review of the literature, on this topic.

## 1. The Epididymis Plays a Key Role in Sperm Maturation

At the end of spermatogenesis, sperm cells are immotile or display a finely twitching movement, are functionally immature, and are unable to fertilize an oocyte. It is during the epididymal transit that they interact with locally synthesized proteins, undergo a series of biochemical and structural changes, and finally acquire progressive motility. Sperm surface modifications occur during epididymal transit through interactions with epididymal secretions, including an increase in the total negative surface charge, modifications in lectin-binding properties, changes in phospholipid composition and in surface glycoproteins, and surface antigen relocalization, all essential for the acquisition of sperm fertilizing ability [1].

The epididymis is a convoluted tubule that connects the testicular efferent ducts, which arise from the rete testis, to the vas deferens, and is composed by the caput, the corpus, and the cauda. It is formed by a pseudostratified epithelium composed of principal cells, clear cells, narrow cells, and basal cells, which forms the blood–epididymis barrier. Narrow and clear cells express the vacuolar proton pump V-ATPase necessary for intraluminal acidification [2]. Basal cells reside at the base of the epithelium along the entire organ (caput, corpus, and cauda): they are thought to be involved in the protection of the epididymis against reactive oxygen species [2]. Principal cells participate in the transepithelial transport of water, solutes, ions, and protein secretion and, together with clear cells, release epididymosomes, the extracellular vesicles that play an important role in the post-testicular maturation of spermatozoa [3].

The efferent ducts are responsible for the 90% of the absorption of the fluid that leaves the rete testis, which involves ion transporters and aquaporin channels and results in an increased sperm concentration in the semen; fluid reabsorption is completed during the transit through the remaining parts of the epididymis, so that the sperm concentration in cauda epididymis is greatly increased compared to that in the rete testis [4]. Sperm concentration by means of fluid absorption is regulated by estrogens: estrogen receptor alpha is highly expressed in the efferent ducts and corpus epididymis in rodents and in efferent ducts in humans [2].

The caput epididymis is the most metabolically active part of the epididymis, since its cells secrete up to 80% of the proteins found in the lumen. In this section of the epididymis, sperm cytoplasmic droplets migrate to the intermediate piece of the flagella by means of clusterin, the most abundant luminal epididymal protein [5].

The cauda epididymis is responsible for the storage of mature sperm: sperm are maintained in a quiescent state due to factors secreted by the local epithelial cells, and their metabolic activity increases 3–5-fold after ejaculation [6]. Sperm transit along the human epididymis is thought to be shorter, and the sperm reservoir capacity very limited, compared to what has been found in other animals; the sperm reservoir capacity does not exceed the number of male gametes required to produce two to three semen samples [1].

As previously mentioned, sperm cells are mostly immotile when they reach the caput epididymis. A key signaling event responsible for sperm motility acquisition is the sperm phosphoprotein phosphatase 1 (PP1) activity: PP1 activity is high in caput epididymis (where sperm are immotile) and inactive in the cauda, where sperm acquire motility [7]. PP1 activity is regulated by PP1 regulatory subunit 2 (PPP1R2), which is activated through its phosphorylation by glycogen synthase kinase 3 (GSK3). GSK3 activity is six times more active in caput than in caudal sperm [7]. In addition to PPP1R2, other phosphatase inhibitors (PPP1R7 and PPP1R11) seem to be involved in the regulation of motility: indeed, their binding to PP1 changes during the sperm transit through the epididymis, so that in caudal sperm, all are bound to PP1 to render it inactive, while in caput sperm, the phosphorylation of PPP1R11 prevents the binding of PPP1R7 to PP1, resulting in PP1 catalytic activity (reviewed in [8]).

Sperm motility is fueled by glycolysis. Sperm have distinct glycolytic isoenzymes and express specific isoforms of proteins essential for glycolysis, but during the transit through the epididymis, they interact with extracellular vesicles secreted by the epididymal epithelium, the epididymosomes, that transfer proteins known to promote motility [8]. In addition, extracellular vesicles found in the human seminal plasma have been found to contain glycolytic enzymes able to produce ATP when supplied to a substrate, as well as glucose transporters, such as GLUT3, GLUT5, and GLUT14, and adenosine triphosphatases [9], and are thought to transfer their glycolytic enzymes to sperm to promote motility, protein tyrosine phosphorylation, and fertilization.

Epididymosomes are also involved in the recognition and removal of defective sperm: two classes of epididymosomes have been identified: CD9-positive and epididymal sperm-binding protein 1-enriched, which bind live and dead sperm, respectively [10]. Epididymosomes also transfer noncoding RNAs (microRNAs, piwiRNAs, and tRNA derived from small RNAs) to the sperm: sperm isolated from the proximal epididymis display, in fact, different noncoding RNA profiles than sperm isolated from the distal epididymis [11]. Such noncoding RNAs are involved in key functions, including transposon silencing and epigenetic inheritance.

Finally, during their transit through the epididymis, sperm acquire factors necessary for binding and penetrating the zona pellucida of the oocyte, namely, those proteins (hCRISP1, Binder of Sperm, ADAM family, etc.) that interact with oligosaccharides on the oocyte membrane [6].

It may easily be inferred from the data presented in this section that epididymal pathologies may significantly impair the sperm fertilizing ability. However, as detailed in the following sections, the role and importance of epididymal pathology as a cause of male factor infertility has been neglected for decades.

## 2. Historical Background

In the 1970s and 1980s, the field of male factor infertility was still in its infancy: the differential diagnosis between obstructive (OA) and non-obstructive azoospermia (NOA) was entrusted to the surgical exploration of the scrotum, testicular biopsy, and eventually, vasovesiculography when a physical exam could not be of help. Testicular biopsy was also recommended in patients with oligozoospermia (sperm count lower than 10 million/mL) displaying normal serum follicle-stimulating hormone (FSH) levels to classify the severity of spermatogenic dysfunction according to the histopathological diagnosis.

**Figure 1:**
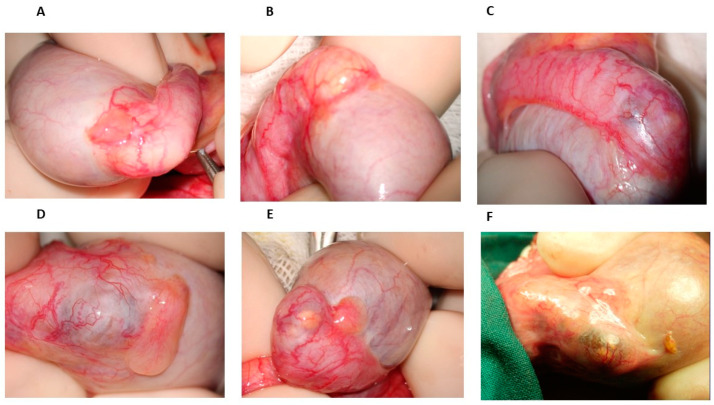
The epididymis at scrotal inspection. (**A**) Normal epididymis. (**B**) Group of yellow dilated tubules appearing under the serosa of the head of epididymis; a grey-whitish area, due to a scar tissue, is visible behind them. (**C**) Small bluish area inside the head of the epididymis. (**D**) Head of the epididymis with a large bluish-dark area, partially covered by whitish scar tissue: some ochre tubules appear under the serosa. (**E**) Head of the epididymis with a bluish-dark area with two tiny yellowish spots inside: caudally, at its end, a whitish scar area is visible. (**F**) Head of the epididymis consisting of half of a dark area with dilated tubules, including a smaller area of yellow tubules.

From 1976 onwards, many patients with azoospermia or severe oligozoospermia referred to our center to undergo testicular biopsy; when the diagnosis of OA was obtained, vasovasostomy or epididymovasostomy was offered. During scrotal surgical exploration, the patterns of epididymal obstruction, with which we became familiar, were characterized by dilated and yellowish tubules proximal to the obstruction site, the latter appearing as a whitish, fibrotic area (Figure 1B) or, in some cases, as a darker one. However, in some cases of men with severe oligozoospermia undergoing testicular biopsy, the inspection of the epididymis revealed some bluish areas of variable size, sometimes isolated (Figure 1C) but otherwise detectable in more points of the head and of the body, sometimes accompanied by yellowish, dilated tubules that were easily visible under the epididymal tunic (Figure 1E,F). The location of such areas did not change over time, as we could determine in the case of a further scrotal exploration in the same patients by the comparison of the surgical pictures obtained in the first and second surgical attempts. Since spermatogenesis was not affected in these patients, according to testis histology reports, we hypothesized that such bluish areas could be the site of an incomplete obstruction of the epididymis leading to oligozoospermia, particularly when ochre tubules with a variable size were detectable in them (Figure 1D).

It was very challenging to find references in the literature of the time to support such a hypothesis. The authors agreed that bluish or white sclerotic tissue indicated epididymal obstruction [12,13,14], but the possibility that a partial epididymal obstruction could lead to severe oligozoospermia was found not to be convincing [15]. Nevertheless, since 1972, one author, Robert Schoysman, dedicated much of his work to demonstrating the role of epididymal pathology in the pathogenesis of male infertility. He was convinced that a partial obstruction of the epididymis could lead to severe oligozoospermia, which could also be amenable to vasoepididymostomy [16]. In a publication, the author presented an illuminating description of this pathology “Once the inflammatory process causing the obliteration through post-inflammatory sclerosis has healed, tubular dilatation upstream of the lesion is not uniform. It starts in the vicinity of the obliterating structure and proceeds in retrograde direction until the whole head of the organ becomes swollen… below the serosa, the tubules are clearly dilated and buff colored. Under the microscope the epididymal tubule near the stenosis is dilated and phagocytosis of the spermatozoa accumulated in excessive number is observed. Since spermatogenesis goes on, this progressive engorgement of the epididymis gives rise to ever increasing pressure. Eventually, rupture of epididymal tubules with escape of sperm into the interstitial tissue may occur giving rise to the formation of spermatic granulomata or spermatorrhagias” [17]. This was accompanied by a didactic histological illustration. In a further contribution, the same author clarified that the final event of the obstructive process is the development of spermatic granulomas: “The development of spermatic granulomas is the final event. If this process takes place deep within the organ, one is usually not aware of it, although careful palpation occasionally allows to discover the hard nodules; but when the granuloma formation is located just beneath the serosa, a brownish coloration of variable diameter may be seen. Ultimately, when the granuloma itself is invaded by vessels and is progressively resorbed, a new sclerotic area appears and thus a new block. Upon the inspection of the epididymis, the area of sclerotic granuloma has a bluish aspect and feels rather hard” [18]. One year later, he delivered a lecture at the Second International Congress on Andrology in Tel Aviv, during which he claimed that epididymal pathology could be a potentially recognizable cause of up to 20% cases of male subfertility when spermatogenesis was not affected, that such pathology could cause both incomplete and complete obstruction, consequently leading to oligospermia as well as to azoospermia, and that not considering the epididymis while performing testicular biopsy could result in the loss of at least half of the value of scrotal exploration [19].

The hypotheses of Schoysman were further confirmed by few other authors. In 1981, Silber and Rodiguez-Rigau demonstrated that 10% of oligozoospermic patients had a partial epididymal obstruction, as demonstrated by a normal testis biopsy [20]. In 1995, Hauser et al. found that oligozoospermic men with normal serum FSH levels and testis volume showed evidence of partial epididymal obstruction at scrotal exploration, coupled with normal spermatogenesis at the testis biopsy, in 81.2% of cases, with surgical correction being able to restore sperm count in 50% of them [21]. Finally, in 1998, Belmonte and Martin de Serrano found that oligozoospermia was due to a partial obstruction in 61% of cases [22]. But, indeed, it was the work of such a visionary and innovative researcher that supported us in becoming confident that the above-described epididymal patterns, often found by us during scrotal exploration in patients with severe oligozoospermia, could indeed represent the macroscopical manifestations of a partial obstruction of the epididymis. This was also confirmed by the testis histology reports, showing mild hypospermatogenesis to normal spermatogenesis, and by the results of the seminal tract washout test, a diagnostic procedure set up by our group [23]: when vasovesiculography showed the distal seminal tract perviousness, the presence of few or no sperm in the urine samples retrieved from the bladder was confirmative of partial or total obstruction, respectively, while a normal sperm count was found when the epididymis was not affected.

The possible clinical conditions that may lead to partial or complete epidydimal obstructions include infections, most of which are asymptomatic, and scrotal trauma. While the testis is covered and protected by the albuginea tunic, and it is functionally divided into several lobules, so that scrotal traumas may result in modest sub-albuginea hematomas that would be reabsorbed or, at most, in the sclerosis of the more peripheral portion of one lobule, which would not significantly affect spermatogenesis, the epididymis is enclosed by a thin serous membrane, and it is made by few conical lobules derived from the enlarged and convoluted terminal portions of the efferent ducts, which constitute the epididymis head, and then open into a single tubule that constitutes the corpus and cauda. Therefore, a traumatic lesion of the epididymis can result in a more severe impairment of the sperm output.

## 3. Partial Epididymal Obstruction May Lead to Oligozoospermia: Our Personal Experience

In the first years of the 1980s, the scrotal ultrasound (SU) become available as a diagnostic tool in the hands of the urologist; however, apart from a study describing persistent ultrasonographic alterations (enlargement of the gland, development of cysts, and inhomogeneous echo pattern) in the epididymis of 45% of vasectomized males [24], the role of epididymal sonography in the diagnostics of male factor infertility remained neglected for many years, or was considered as of limited clinical value [25,26,27,28,29]. From 1988, we sought to determine the diagnostic accuracy of SU in detecting partial epididymal obstructions in patients with oligozoospermia. To exclude a potential source of bias, all patients were evaluated by the same operator and following ejaculation (patients underwent semen analysis on the same day). The normal ultrasonographic pattern of the caput epididymis were established by earlier studies [30,31] as homogeneous and isoechoic with the testis parenchyma, while the sonographic characteristics of the gland were judged as “altered” when hyperechoic areas or calcific spots, hypoechoic areas, cysts, and microcysts were found, and as an ‘inhomogeneous echo pattern’ when the concomitant existence of different sonographic anomalies was evident.

The first ten subjects studied (31–45 years old) had bilateral absence of the vas deferens. SU demonstrated bilateral epididymal alteration in all cases: an inhomogeneous echo pattern in 12, hyperechoic areas or spots in 4, and hypoechoic areas in 4. A further surgical exploration showed whitish or yellowish dilated tubules in 2 epididymes; dilated tubules, dark ochre slightly narrow tubules, spermatorragias, whitish fibrosis, and microcysts in 14; and spermatorragias with fibrosis and (micro)cysts in 4 [32]. Subsequently, we evaluated 35 patients with suspected OA: 65 out of 70 epididymes presented an inhomogeneous echo pattern, while at surgical inspection, all showed signs of obstruction [33]. Two years later, we reviewed the data of thirty normozoospermic patients undergoing surgery for hydrocele repair, risk of funicular torsion, or chronic orchialgia of unknown origin, with a total of 45 epididymes being surgically inspected. The SU per-formed before surgery revealed a normal epididymal echo pattern in 36 and an altered one in 9, with surgical exploration confirming these reports in 34/36 and in 9/9 cases. Epididymal alterations were described as cysts, tubules alterations, and spermatorragias, with only modest signs of partial obstruction being found [34]. Another study was performed in 144 non-azoospermic infertile men, with normal serum FSH levels and a testicular volume higher than 10 mL, who underwent testis biopsy or testicular exploration prior to vasovesiculography or seminal tract washout. The comparison between scrotal exploration and SU was made possible for 65 epididymes: epididymal abnormalities were found in 49 and 52 cases by scrotal exploration and SU, respectively, while a normal epididymal structure was found by SU in 13 cases but confirmed by scrotal exploration only in 5 cases [35]. The results of these four studies are summarized in Table 1; as it may be inferred by the data presented, the SU evaluation was very sensitive in detecting epididymal abnormalities.

Once the diagnostic accuracy of SU in detecting epididymal abnormalities was confirmed, we evaluated some cohorts of infertile men to determine the prevalence of epididymal pathologies in this subset of patients. The evaluation of 265 randomly selected infertile subjects (median age 33.5 y, range 21–47) confirmed that epididymal pathology could be involved in the pathogenesis of idiopathic oligozoospermia, since 245 out of 518 epididymes (47.2%) evaluated by SU revealed altered echo patterns [36]. The SU evaluation of 259 consecutive infertile patients revealed unilateral and bilateral epididymal alterations in 70 (27.0%) and 78 (30.1%) cases, respectively [37]. A large sample size, controlled study, evaluating 612 infertile men and 293 normozoospermic controls without history or signs of sexually transmitted diseases or male accessory gland infection, demonstrated epididymal alterations at SU in 54.9% vs. 19.4%, respectively (*p* < 0.0001) [37]. The percentage of SU abnormalities found in the healthy controls was lower than the one reported by Leung [30] in an older cohort of asymptomatic men.

The impact of such epididymal abnormalities on sperm parameters was evaluated by us in a randomized clinical study involving 98 patients with a normal epididymal echo pattern (group A) and 98 age-matched (mean age 34.7 ± 4.4 y; range 28–49 y) subjects showing bilateral epididymal alterations at SU (group B). The inclusion criteria were testis volume higher than 10 mL and normal serum FSH level to exclude patients with sperm parameter alterations attributable to spermatogenic dysfunction. Sperm count and total motile count were significantly lower in group B compared to group A (74.9 vs. 108.8 million, *p* = 0.0003, and 10.5 vs. 14.1 million, *p* < 0.05, respectively), while sperm morphology was not affected [37]. We also had the possibility of evaluating the histopathologic abnormalities associated with epididymal inhomogeneous echo patterns in 16 patients undergoing surgery for orchidectomy, epididymovasostomies, and micro-surgical epididymal sperm aspiration. Histology showed the concomitant presence of dilated and stenotic (bottleneck) epididymal tubules with signs of inflammation in all cases, lipofuscine-like pigment infiltration in tubular epithelial cells (nine cases), and hemosiderine accumulation in interstitial macrophages (three cases) [37].

The results of these studies are compelling both for the predictive ability of SU in detecting epididymal abnormalities, possibly leading to epididymal sub-obstruction and oligozoospermia, and for the key role that epididymal pathology may play in the pathogenesis of oligozoospermia when spermatogenesis is not affected. The minimal discrepancy between the SU and scrotal exploration findings could be easily explained by the intrinsic limitation of the SU technique, which is unable to detect anatomic alterations when two contiguous areas have a similar ultrasound density. Interestingly, most of the epididymal abnormalities found in oligozoospermic men (about 50% of cases) were not detectable by palpation. What is also noteworthy is that epididymal abnormalities were found also in 20% of normozoospermic men, but they were not severe enough to affect the sperm output.

## 4. Scrotal Ultrasound as a Diagnostic Tool for Epididymal Pathology in the Literature: Better Late than Never

The first mention of an epididymal pathology being diagnosed by SU was by Carroll and Gross, who reported that SU could identify patients with epididymitis and spermatoceles [38]. In the same year, Jequier et al. first recognized partial epididymal obstruction as a cause of oligozoospermia, which preceded the development of obstructive azoospermia in 8 out of 71 patients [39]. Patel and Parek found 37 cases of epididymitis in a cohort of 124 subjects, although they could not report any “obvious relation between oligospermic and azoospermic patients with scrotal sonographic findings except for testicular atrophy” [27]. Nashan et al. found cystic or “fibrotic conversions after healed inflammation” when a thickened epididymis was found at palpation [28]. Hamm, however, was skeptical about the diagnostic application of SU in the case of epididymal pathology: he wrote that “the echogenicity of the epididymis … may occasionally be increased (due to additional hemorrhages)”, but a “successful therapy will lead to a reduction in size, and, in most cases, the epididymis will regain its normal shape, size, and echogenicity”, that “the diagnosis of chronic epididymitis by sonography is a ‘white lie’, since this diagnosis is established on the basis of the clinical findings after exclusion of other pathologies”; and that “the diffuse, chronic-inflammatory alterations of the epididymis are barely detectable on ultrasound; the only change that may be seen is moderate enlargement of the organ” [29]. Still in 1999, the evaluation of 1372 infertile men by means of SU revealed abnormal findings in 38% of them, but no epididymal alteration apart from spermatoceles were found [40].

In the first decade of the present millennium, however, more authors focused their attention on epididymal pathology. Moon et al. described epididymal tubule ectasia as “multiple anechoic tubular or round structures representing the dilated epididymal duct” [41]. Isidori and Lenzi reported that a long-standing obstruction of the epididymal tubules could be associated with a diffuse dilation of the efferent ducts and enlargement of the epididymal body, which appeared hypoechoic, and described the chronic epididymitis as resulting from the incomplete treatment of epididymoorchitis or irreversible structural alterations secondary to chronic inflammatory changes and granulomatous reaction [42]. Moon and Kim suggested the use of SU to assess the possible obstruction site as well as the presence or absence of chronic epididymitis [43]. More recently, a systematic review on the diagnostic accuracy of SU in the evaluation of the infertile men clarified that the epididymis head and/or tail dilation is suggestive of obstruction or inflammation of the male genital tract, both being related, along with abnormalities in the echo pattern, to impaired sperm parameters [44]. Finally, in 2021, Lotti et al. provided a detailed description of the epididymal alterations that could be evaluated by SU: “in the chronic form (of the epididymitis) the epididymis is often dilated and may appear hyperechoic and vascularization is only slightly increased. A dilated epididymis associated with echo pattern abnormalities (including calcifications) may also represent the outcome of a past infection/inflammation, currently asymptomatic. On the other hand, in subjects with obstructive azoo- or oligospermia, the detection of epididymal enlargement may suggest post-testicular obstruction, which could be (i) at the epididymal level (especially when the downstream vas deferens shows a normal size), (ii) at the vas deferens level, especially in men treated by epididymovasostomy or after vasectomy or (iii) at the prostatic level, the latter to be further investigated extending ultrasonography to the prostate–vesicular region. Furthermore, ultrasonography allows the assessment of epididymal nodules, often perceived at physical examination, frequently represented by cysts, but possibly underlying benign (including tuberculosis-related granulomatous masses) or, very rarely, malignant lesions. Finally, ultrasonography is useful in imaging epididymis after scrotal trauma, often showing features mimicking epididymitis” [45].

## 5. Concluding Remarks

The intent of the present scientific contribution was to report our clinical experience about the role of partial epididymal obstruction as a cause of (otherwise) idiopathic oligozoospermia and, at the same time, to celebrate Robert Schoysman, an innovative and visionary researcher and surgeon who described for the first time such a hypothesis, but whose work was not appreciated by his contemporaries, which caused his suggestion to be ignored for decades.

The value of our data comes from the comparison between the SU findings and the visual inspection of the epididymis, the latter being made possible during scrotal exploration, which was justified even in patients with oligozoospermia, at that time, by diagnostic and therapeutic interventions at present considered obsolete and no longer feasible in light of the current guidelines. Since these data were presented at international meetings but never published in peer-reviewed journals, we felt the need to report them in the present article. The conclusions we can draw from our experience are the following:

- Anatomical irregularities of the epididymis secondary to chronic inflammation (epididymitis) or traumas may interfere with sperm transit;
- Partial obstruction of the epididymis is not an uncommon finding in men with oligozoospermia when testis volume and serum FSH levels are normal: a timely and accurate diagnosis may be, therefore, of great importance in order to provide the appropriate treatment and avoid unnecessary hormonal treatments designed to enhance sperm production;
- SU abnormalities of the epididymis are highly predictive of anatomic alteration of the gland (sensitivity of 81.8 to 100% and specificity of 62.5 to 100%); therefore, SU could be of help in the management of infertile men with oligozoospermia.

Epididymal pathology affecting infertile men has only recently received some attention in studies, as detailed above. However, further research should be conducted in this area to improve our understanding of the role of epididymal pathology in the pathogenesis of oligozoospermia and to identify opportune treatment options for this challenging pathological condition.

## Figures and Tables

**Table 1:** Comparison of epididymal patterns during scrotal inspection and at scrotal ultrasound.

		Epididymal Pattern During Scrotal Exploration	
*Cohorts Studied*	Epididymal Pattern at US	Normal	Abnormal
10 with CBAVD (20 epididymes) [21]	Normal	0	0
	Abnormal	20	20
*Sensitivity: 100%, Specificity: N.A.*			
35 with OA (70 epididymes) [22]	Normal	0	5
	Abnormal	65	65
*Sensitivity: 92.86%, Specificity: N.A.*			
30 normozoospermic (45 epididymes) [23]	Normal	34	2
	Abnormal	0	9
*Sensitivity: 81.82%, Specificity: 100%, Ref.*			
144 infertile men (65 epididymes) [24]	Normal	5	8
	Abnormal	3	49
*Sensitivity: 85.9%, Specificity: 62.5%; Ref.*			

CBAVD = congenital bilateral absence of vas deferens. OA = obstructive azoospermia N.A. = not applicable (specificity cannot be computed when disease prevalence = 100%).

## Data Availability

No new data were created.
